# Electrophysiological Cross-Language Neighborhood Density Effects in Late and Early English-Welsh Bilinguals

**DOI:** 10.3389/fpsyg.2012.00408

**Published:** 2012-10-18

**Authors:** Giordana Grossi, Nicola Savill, Enlli Thomas, Guillaume Thierry

**Affiliations:** ^1^State University of New York at New PaltzNew Paltz, NY, USA; ^2^University of YorkYork, UK; ^3^Bangor UniversityBangor, UK

**Keywords:** bilingualism, ERPs, neighborhood density, reading, orthography

## Abstract

Behavioral studies with proficient late bilinguals have revealed the existence of orthographic neighborhood density (ND) effects across languages when participants read either in their first (L1) or second (L2) language. Words with many cross-language (CL) neighbors have been found to elicit more negative event-related potentials (ERPs) than words with few CL neighbors (Midgley et al., 2008); the effect started earlier, and was larger, for L2 words. Here, 14 late and 14 early English-Welsh bilinguals performed a semantic categorization task on English and Welsh words presented in separate blocks. The pattern of CL activation was different for the two groups of bilinguals. In late bilinguals, words with high CLND elicited more negative ERP amplitudes than words with low CLND starting around 175 ms after word onset and lasting until 500 ms. This effect interacted with language in the 300–500 ms time window. A more complex pattern of early effects was revealed in early bilinguals and there were no effects in the N400 window. These results suggest that CL activation of orthographic neighbors is highly sensitive to the bilinguals’ learning experience of the two languages.

## Introduction

Research over the last 20 years has shown that, within a language, the number of neighbors (i.e., words created by changing a single letter of a target word – Coltheart et al., 1977) of a target stimulus influences the processing of the target. This effect, named the neighborhood density (ND) effect, is modulated by several factors. For example, whereas words with a high number of neighbors are generally recognized faster than words with a low number of neighbors in lexical decision tasks, an inhibitory effect has generally been found with non-words (e.g., Coltheart et al., 1977; Andrews, 1989; Holcomb et al., 2002). With words, the effect is also modulated by the frequency of the target (e.g., Andrews, 1989, 1992) and the relative frequency of the neighboring words compared to the frequency of the target words (longer RT when neighbors have a higher frequency than the target; see Perea, 1998, for a review). Finally, different ND effects have been observed in different tasks. For example, Carreiras et al. (1997) found that ND effects were inhibitory in a progressive demasking task (where participants had to identify the stimuli), null in a lexical decision task, and facilitatory in a naming task.

Electrophysiological studies have investigated neural indices of such effects. Holcomb et al. (2002) showed that the N400, a marker of lexical and semantic processing usually observed between 350 and 500 ms (e.g., Kutas et al., 2006), was larger when targets had a high compared to low ND. This effect was found in both a lexical decision (for both words and non-words) and semantic categorization task, which suggests that similar mechanisms are at work in the two tasks, and was recently replicated by Müller et al. (2010) and Laszlo and Federmeier (2011). The larger N400 to targets with high, compared to low, ND has been interpreted in terms of increased lexico-semantic activation of, and competition among, neighbors, according to Holcomb and colleagues, and increased semantic activation of neighbors according to Laszlo and Federmeier (2011). Because ND effects in the N400 time window have been found for both words and pseudowords, Laszlo and Federmeier have concluded that access to meaning is attempted regardless of the orthographic status of the target. According to the authors, these data therefore argue against staged models of word recognition (e.g., Forster, 1999) and support cascade models (e.g., Harm and Seidenberg, 2004).

Both behavioral and electrophysiological studies have shown that ND effects can also be observed cross-linguistically. For example, in van Heuven et al.’s (1998) first experiment, proficient Dutch-English bilinguals performed a progressive demasking task on both Dutch (L1) and English (L2) words. Identification speed in both languages was negatively influenced by the number of orthographic neighbors in the other language (i.e., the higher the ND, the longer the RT). In Experiment 4, a different group of proficient Dutch-English bilinguals performed a lexical decision task on English (L2) words. Again, RTs were longer for English words that had a high number of neighbors in Dutch (L1). These and other data (e.g., Alternberg and Cairns, 1983; Frenck-Mestre, 1993; Bijeljac-Babic et al., 1997) suggest that orthographic representations for the first and the second languages might be organized together in highly proficient bilinguals and trigger a complex series of activation and inhibition processes among words belonging to different languages (Dijkstra and Van Heuven, 2002).

The N400 modulation by ND has also been observed cross-linguistically (Midgley et al., 2008). In a categorization experiment, late French-English bilinguals, all proficient in L2 (English), were asked to perform a go/no-go task and press a button when an animal name was presented on the screen. Participants were presented with two separate lists (French and English words) whose order was counterbalanced across subjects. Cross-language (CL) ND was manipulated in the following way: 50% of the French words had a high number of neighbors in English and 50% had a low CLND. Similarly, 50% of the English words had a high number of neighbors in French and 50% had a low CLND. In general, event-related potentials (ERPs) were more negative for targets with high, compared to low, CLND. However, the pattern of effects depended on the target language. The N400 (300–500 ms) effect peaked later and was less widely distributed for L1 than L2 targets. Furthermore, early effects (P2/N2, 175–275 ms) were present only for L2 targets. These effects were absent in a group of monolingual English speakers.

Midgley et al. (2008) interpreted the difference in CLND effects between the two languages in terms of frequency of exposure: the participants were more proficient in French, French being their first language; therefore, the connection strength between lexical representations was stronger for L1 than L2. As a consequence, French neighbors were more easily activated by English targets than English neighbors by French targets. A similar interpretation was proposed to explain the presence of early effects (P2/N2) for L2 targets (which were present in Holcomb et al., 2002, but only in the categorization task). According to the authors, differences in frequency between the targets and their neighbors in the two studies would explain the discrepancy in results. In Holcomb et al. (2002), both target and neighboring words had a high subjective written frequency, whereas, in Midgley et al. (2008), L2 targets had a lower subjective frequency than their L1 neighbors. Therefore, in the second study, the activation and competition from high-frequency neighbors would have started earlier.

### Goals of the present study

The behavioral and electrophysiological data reviewed so far support the non-selective access hypothesis, according to which, during presentation of single words, multiple lexical representations are activated (mainly bottom-up); especially those representations from L1 that have some sort of orthographic, phonological, or semantic overlap with L2 input (e.g., van Heuven et al., 1998; Dijkstra et al., 1999; Haigh and Jared, 2007; for activation through translation, see Thierry and Wu, 2007; Wu and Thierry, 2010; Zhang et al., 2011). According to the Bilingual Interaction-Activation (BIA+) model (Dijkstra and Van Heuven, 2002), these multiple representations compete with each other through lateral inhibition. As a result, both within-language and CL lexical interference effects can arise. A similar interpretation was proposed by Holcomb and Grainger (2007) to explain Holcomb et al.’s (2002) data on within-language ND effects. Midgley et al. (2008) also interpreted their CL effects in terms of lexical competition between word form representations (orthographic and/or phonological) from the two languages.

The goal of the present study was to replicate and extend Midgley et al.’s (2008) experiment by employing a different language pair (English and Welsh) and two groups of bilingual individuals: late bilinguals, who started learning Welsh during or after puberty, and early bilinguals, who learn both English and Welsh early in life. The comparison between late and early bilinguals will provide invaluable information on whether the pattern of CL activation differs depending on when the second language is learned: consecutively to, or concurrently with, the first language. Studying Welsh and English as a language pair allows testing potential interactions between orthographic transparency and language non-selective lexical access.

Welsh orthography is rather different from English orthography. First, it is transparent and, in contrast to English, has essentially one-to-one mapping between graphemes and phonemes (Frost et al., 1987; Ellis and Hooper, 2001). Also, it is characterized by letter combinations fairly uncommon in English. For example, many words start with double consonants such as “ll”/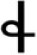/and “ff”/f/. Diphthongs like “wy”/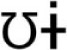/and “ae”/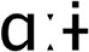/or/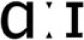/ are quite common; and “w”/u/and “y”/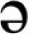/ are vowels. Therefore, Welsh word forms can look quite different from English word forms. Indeed, native English speakers who are not familiar with Welsh show no word and pseudoword superiority effects (considered to be measures of familiarity with the words and the orthography of a language, respectively; McClelland, 1976; Carr and Pollatsek, 1985; Grainger et al., 2003) in a forced-choice letter identification task (Grossi et al., 2008).

Participants performed a semantic categorization task with Welsh and English words presented in separate blocks. Based on previous literature on within-language and CLND, it was predicted that high, compared to low, CLND words would generate more negative ERPs starting at around 175 ms post-stimulus onset. Based on Midgley et al. (2008), this effect was predicted to be asymmetric in late bilinguals, with stronger effects for L2 compared to L1 targets, assuming that different pattern of early and late effects for L1 and L2 in late proficient bilinguals reflects frequency of exposure. In early bilinguals, based on the frequency of exposure hypothesis, we predicted similar effects for L1 and L2 targets, as these participants had extensive exposure to both languages.

## Materials and Methods

### Participants

A detailed description of participants’ characteristics can be found in Grossi et al. (2010); see also Table 1, p. 126)^1^. Analyses were carried out on 14 early Welsh/English bilinguals (six females, mean age of 38.4 years, range 22–52 years) and 14 late learners of Welsh (10 females, mean age of 40.3 years, range 25–52 years). Based on self-report, all participants had normal or corrected-to-normal vision (20/20), and none had a history of neurological disorders. Based on self-report and the Edinburgh Handedness Inventory (Oldfield, 1971), all late bilinguals were right-handed; in the early bilingual group, 12 participants were right-handed, one was left-handed, and one was ambidextrous. All participants were paid £7/h for their participation.

Based on self-reports, early bilinguals learned Welsh from birth (*n* = 10) or early in life (three from age 3, and one from age 5); as for English, seven learned it from birth, two before age 3, three from age 4, and two from age 5. The primary language spoken at home until 2 years of age was Welsh for six participants, a mix of Welsh and English for four participants, and English for four participants. Elementary education was in Welsh for five participants, balanced for one participant, predominantly in Welsh for six participants, and predominantly in English for two participants. Middle school and high school instruction was in both Welsh and English for all early bilinguals. In terms of language proficiency, all early bilingual participants rated themselves as native-like speakers in both languages. All participants rated themselves as native-like in reading English; for Welsh, eleven participants rated themselves as native-like, and three participants as somewhat proficient. Early participants reported speaking Welsh almost half of the time (*M* = 47.5%, SD = 25.8) and reading Welsh for recreational reading 28% of the time (SD = 26.7).

For late bilinguals, the mean age of acquisition for Welsh was 28.3 years (SD = 8.7), and the average number of years of Welsh was 11.9 (SD = 6.9). Four participants held a college degree, and 10 held a post-graduate degree. The primary language spoken at home until 2 years of age was English for 13 participants, and Polish for 1 participant. Elementary education was in English for all participants. Most participants had English as the only language of instruction in both middle school (*n* = 11) and high school (*n* = 12; the other participants were exposed to some Welsh). When asked to indicate how well they felt they spoke Welsh and English, all participants rated themselves as native-like in English; nine participants rated themselves as native-like in Welsh, four as somewhat proficient, and one between these two levels. In terms of proficiency in reading, all participants rated themselves as native-like in English; eight participants rated themselves as native-like in Welsh, five as somewhat proficient, and one as low proficient. Participants reported to speak Welsh 30% of the time (SD = 22.3) and to read Welsh for recreational reading 22.5% of the time (SD = 14.8).

Proficiency in Welsh was also measured objectively with a translation task including all Welsh words used in the semantic categorization task (*n* = 96). The task was administered at the end of the experimental session before the debriefing. Participants were asked to circle all the familiar Welsh words and, when possible, provide the correct English translation. As expected, early bilinguals translated Welsh words with a higher degree of accuracy (91.15 vs. 80.73%) than late bilinguals and indicated fewer Welsh words as being completely unfamiliar (3.57 vs. 11.24%; see Grossi et al., 2010, Table 2, p. 126 for more information).

### Stimuli and materials

Two lists of 80 Welsh and 80 English words were created: 50% with high CLND and 50% with low CLND. Therefore, there were 40 words in each of the following categories: high CLND Welsh, low CLND Welsh, high CLND English, low CLND English. In addition, animal names were used as probe stimuli (20% per block, *n* = 16 for each language block). Welsh words were selected from the Cronfa Electroneg o Gymraeg (Ellis et al., 2001); English words were selected from the CELEX database (Baayen et al., 1995). Words were four- or five-letter words, either mono- or bi-syllabic. Words with at least one occurrence per million were selected and used to calculate the number of orthographic neighbors of words within and across languages. The final set of stimuli for the study were 80 English (mean frequency = 80.32, SD = 93.92) and 80 Welsh words (mean frequency = 74.85, SD = 70.81; the difference in frequency was not significant, *p* = 0.69) between four and five letters in length with half of the items in each language having many orthographic neighbors in the other language and the other half having few neighbors in the other language. English items with high Welsh ND had a mean number of Welsh neighbors of 7.9 (range = 4–12, SD = 2.1). English items with low Welsh ND had 0.23 (range = 0–2, SD = 0.58) neighbors on average. The difference between the two means was significant (*p* < 0.0001, two-tailed). Stimuli were matched on within-language neighborhood size. The list of stimuli and information about orthographic and lexical characteristics can be found in Grossi et al. (2010).

The 16 Welsh and 16 English animal names were matched in length (Welsh, *M* = 4.5, SD = 0.52; English, *M* = 4.43, SD = 0.51; *p* = 0.73, two-tailed) and frequency (Welsh, *M* = 26.56, SD = 41.64; English, *M* = 15.63, SD = 29; *p* = 0.4, two-tailed).

### Procedure

Participants gave written consent and filled out the handedness and biographical questionnaires. Next, they performed the semantic categorization task. All participants were tested in a sound-attenuating and electrically shielded booth, and seated 100 cm directly in front of a 19-inch monitor. The sequence of events was the following: a fixation point appeared at the center of the screen and served as a warning signal that a trial was about to begin; the fixation point was followed by a random and variable interval between 500 and 700 ms, after which words were presented for 1000 ms and followed by 1000 ms of blank screen. Each trial ended with a screen indicating that participants could blink. Participants were instructed to press a button, as quickly and as accurately as they could, every time an animal name would appear on the screen. Practice trials presented at the beginning allowed participants to familiarize themselves with trial structure. The session was self-paced: participants controlled when the next trial would begin by pressing a button on a response box. The entire experimental session lasted between 2 and 3 h.

### ERP data collection

Electrophysiological data were recorded in reference to Cz at a rate of 1000 Hz from 64 Ag/AgCl electrodes placed according to the extended 10–20 convention (Neuroscan system). Impedances were kept below 7 kΩ. EEG activity was filtered on-line band pass between 0.1 and 200 Hz and re-filtered off-line with a 30 Hz low pass zero phase shift digital filter. Eye-blinks were detected using the vertical electrooculogram bipolar channel. Potential variations exceeding a threshold of 20% of maximum EEG amplitude over the duration of a complete individual recording session were automatically registered as artifacts and contributed to the computing of a model blink artifact (derived from more than 100 individual blink artifacts in each participant). Artifacts were then individually corrected by subtracting point-by-point amplitudes of the model from signals measured at each channel proportionally to local maximum signal amplitude. Eye movements, drifts, and other artifacts were removed by an algorithm that eliminated all events associated with brain waves that were larger than 75 μV or smaller than −75 μV. The percentage of accepted trials was 89%. Epochs ranged from −500 to 1000 ms after the onset of the critical word. Baseline correction was performed in reference to pre-stimulus activity (500 ms baseline) and individual averages were digitally re-referenced to the left and right mastoid channels offline. Behavioral data were collected simultaneously to ERP data.

### Measures and analyses

Analyses were conducted in the following time windows: 175–300 and 300–500 ms (classical N400 window). Omnibus analyses were conducted on the following factors: Group (between-subjects), Language (English, Welsh), and CLND (high, low). In order to describe the scalp distribution of Language and CLND effects, the following repeated measures factors were also included: Hemisphere (left, right), Laterality (lateral, medial), and Anteriority (central, centroparietal, parietal). Analyses were informed by regions of interest highlighted by Midgley et al. (2008) and conducted at the sites where CLND effects were largest, based on visual inspection. The following electrodes were included in the main analyses: C3/4, C1/2, CZ (central), CP3/4, CP1/2, CPZ (centroparietal), and P3/4, P1/2, PZ (centroparietal). Analyses on midline sites were run separately from hemisphere analyses.

In late bilinguals, CLND effects started at around 175 ms for Welsh stimuli over central, centroparietal, and parietal sites and continued until approximately 500 ms. In early bilinguals, the largest differences were more frontal. Therefore, for this group, analyses were also carried out over frontal (F5/6, F3/4, Fz) and frontocentral (FC3/4, FC1/2, FCZ) sites. The dependent variable was mean ERP amplitude in each of the intervals of interest. Words rated as unfamiliar by the participants were excluded from analysis. Significant interactions involving condition effects were followed up by simple effects analyses. Adjusted *p*-values (Geisser–Greenhouse correction) are reported for all within-subject measures with more than one degree of freedom.

## Results

### Behavioral results

A detailed discussion of the results can be found in Grossi et al., 2010; see Table 4 on p. 129). Late bilinguals were faster and more accurate in detecting target words in English than Welsh. Mean accuracy was 99.11% (SD = 1.91) for English and 84.15% (SD = 16.65) for Welsh. Mean RTs were 575.96 ms (SD = 74.54) for English and 666.40 ms (SD = 76.14) for Welsh. The difference between language conditions was significant for both RT and accuracy (both *p*’s < 0.01). Early bilinguals showed no differences in accuracy for the two languages (English, *M* = 98.21, SD = 2.66; Welsh, *M* = 94.20, SD = 9.48; *p* = 0.16), and were faster in recognizing English (*M* = 565.07 ms, SD = 64.47) than Welsh (*M* = 619.26 ms, SD = 72.92) targets (*p* = 0.008).

### Event-related potentials

**Figure 1:**
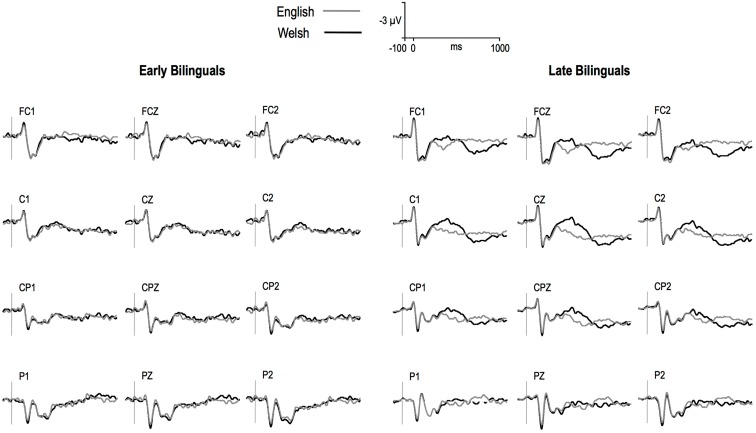
**Mean grand-averages ERPs for Welsh and English stimuli over medial and midline sites in late and early bilinguals**. Negative is plotted up.

**Figure 2:**
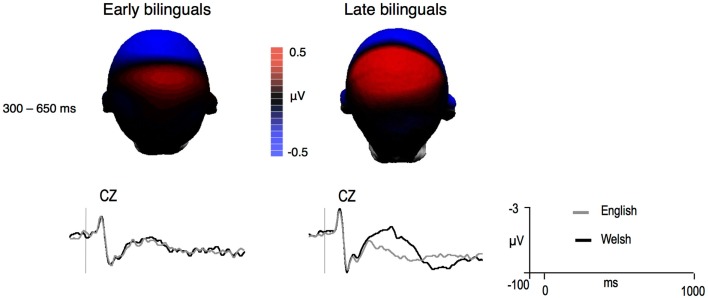
**Difference voltage maps representing the 300–650 ms language effect (English–Welsh) in the two groups of bilinguals and mean grand-averages ERPs at the site where the effect was largest (negative is plotted up)**.

Figure 1 depicts the ERPs elicited by English and Welsh words for the two groups of participants. Welsh targets elicited more negative ERPs than English targets from around 300 ms and until approximately 650 ms for late but not in early bilinguals. The distribution of the Language effect (in terms of difference voltage maps) is shown in Figure 2. Omnibus hemisphere analyses for the two time windows showed that the CLND effect and Language effect differed between groups [175–300 ms: Language × ND × Group, *F*(1,26) = 5.52, *p* < 0.03; ND × Hemisphere × Group, *F*(1,26) = 5.63, *p* < 0.03; ND × Hemisphere × Laterality × Group, *F*(1,26) = 3.94, *p* < 0.06; ND × Hemisphere × Laterality × Group, *F*(1,26) = 12.99, *p* = 0.001; 300–500 ms: Language × Laterality × Group, *F*(1,26) = 3.74, *p* = 0.06; ND × Hemisphere × Laterality × Group, *F*(1,26) = 5.18, *p* = 0.03; Language × Laterality × Anteriority × Group, *F*(2,52) =  5.95, *p* = 0.006]. Table 1 presents a summary of relevant findings at centroparietal sites in an omnibus ANOVAs for the two groups. Only the main results and follow-up analyses will be discussed in the next section.

**Table 1:** **Relevant findings for late and early bilinguals in analyses at central, centroparietal, and parietal sites**.

Late bilinguals	175–300 ms	Hemisphere analysis
		ND, *F*(1,13) = 4.68, *p* = 0.05
		ND × Hemisphere × Laterality, *F*(1,13) = 4.67, *p* = 0.05
		Language × Laterality × Anteriority, *F*(2,26) = 9.73, *p* = 0.001
		Midline analysis
		ND, *F*(1,13) = 9.25, *p* = 0.009
		Language × Anteriority, *F*(2,26) = 3.3, *p* < 0.08
	300–500 ms	Hemisphere analysis
		ND, *F*(1,13) = 4.07, *p* = 0.065
		Language × ND × Hemisphere × Anteriority, *F*(2,26) = 3.25, *p* = 0.056
		Language × Laterality, *F*(1,13) = 10.17, *p* = 0.007
		Language × Laterality × Anteriority, *F*(2,26) = 13.37, *p* < 0.0001
		Midline analysis
		ND, *F*(1,13) = 5.13, *p* = 0.04
		Language, *F*(1,13) = 4.13, *p* = 0.06
		Language × Anteriority, *F*(2,26) = 8.62, *p* = 0.01
Early bilinguals	175–300 ms	Hemisphere analysis
		ND × Hemisphere × Laterality, *F*(1,13) = 8.84, *p* = 0.01
		Language × Laterality, *F*(2,26) = 4.86, *p* = 0.04
	300–500 ms	Hemisphere analysis
		ND × Hemisphere × Laterality, *F*(1,13) = 4.2, *p* = 0.06

*The results pertain to omnibus ANOVAs*.

### Late bilinguals

#### 175–300 ms

**Figure 3:**
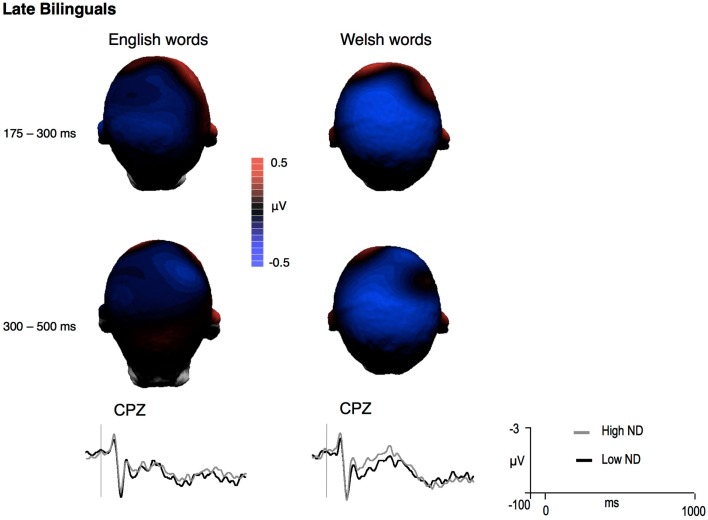
**Difference voltage maps representing the cross-language ND effect (high ND – low ND) in late bilinguals and mean grand-averages ERPs at the site where the effect was largest (negative is plotted up)**.

Analyses conducted on lateral and medial electrodes showed that ERP amplitudes were more negative for high compared to low CLND targets in this time window [*F*(1,13) = 4.68, *p* = 0.05]; this effect interacted with Hemisphere and Laterality. Follow-up analyses showed that CLND was significant as a main effect over the left hemisphere sites [*F*(1,13) = 5.45, *p* < 0.04]; over the right hemisphere sites, ND was significant only over medial sites [ND × Laterality, *F*(1,13) = 9.5, *p* = 0.009; medial sites, *p* < 0.05; over right lateral sites, a significant interaction between ND and Language was observed at the 0.05 level, but analyses carried out separately for the two languages did not reveal any significant ND effect]. Therefore, overall, the CLND effect (more negative ERPs to high than low CLND targets) was more robust over the left hemisphere sites and over the medial sites (Figure 3). This main effect did not interact with Language (all *p*’s > 0.1). These results were confirmed by midline analyses (ND, *F*(1,13) = 9.25, *p* = 0.009; no significant interactions between Language and CLND were observed, all *p*’s > 0.11).

In hemisphere analyses, Language interacted with Laterality and Anteriority, revealing some distributional differences between targets in the two languages. However, follow-up analyses did not reveal any reliable Language effect in this time window (all *p*’s > 0.08). Similarly, midline analyses only revealed a trend for significance for the Language × Anteriority interaction, but no significant Language effects were found when analyses were run at each level of Anteriority (all *p*’s > 0.34).

#### 300–500 ms

In hemisphere analyses, ERPs tended to be more negative for high compared to low CLND targets [*F*(1,13) = 4.07, *p* = 0.065]; this effect was qualified by a four-way interaction with Language, Hemisphere, and Anteriority. For English targets, CLND was not significant as a main effect (*p* = 0.5), but interacted with Hemisphere and Laterality [*F*(1,13) = 7.56, *p* < 0.02]. However, no ND effects were significant in follow-up analyses by Hemisphere and Laterality (all *p*’s > 0.21). In contrast, the ND main effect was significant for Welsh targets [*F*(1,13) = 4.77, *p* < 0.05]. No other interaction between Language and ND reached significance. Analyses over the midline sites revealed a main effect for CLND [*F*(1,13) = 5.13, *p* = 0.04]. This effect did not interact with Language (all *p*’s > 0.2).

Event-related potentials were more negative for Welsh than English targets in this time window. In hemisphere analyses, Language interacted with Laterality and with Laterality and Anteriority. Follow-up analyses showed that the Language effect was significant over medial sites [Language × Anteriority, *F*(2,26) = 6.35, *p* = 0.02; central, *p* = 0.003; centroparietal, Language × Hemisphere, *p* = 0.05; parietal, all *p*’s > 0.14] but not lateral sites (all *p*’s > 0.13). Midline analyses revealed a similar pattern of results: the main effect of Language approached significance [*F*(1,13) = 4.13, *p* = 0.06] and was qualified by a Language × Anteriority interaction [*F*(2,26) = 8.62, *p* = 0.01]: Welsh targets elicited more negative ERP amplitudes than English targets at CZ and CPZ sites (*p* = 0.003, *p* < 0.05, respectively; PZ, *p* = 0.78).

### Early bilinguals

#### 175–300 ms

**Figure 4:**
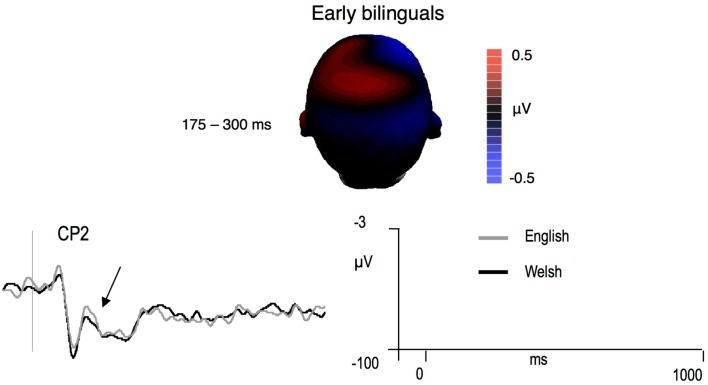
**Difference voltage maps representing the 175–300 ms language effect (English – Welsh) in early bilinguals and mean grand-averages ERPs at the site where the effect was largest (negative is plotted up)**.

Hemisphere analyses over central, centroparietal, and parietal sites revealed an interaction between CLND, Hemisphere, and Laterality [*F*(1,13) = 8.84, *p* = 0.01]. Follow-up analyses carried out on each hemisphere separately did not reveal any significant ND effects. No ND effects were observed over the midline sites (all *p*’s > 0.33). ERP amplitudes were more negative for English than Welsh targets at centroparietal and parietal sites [Language × Anteriority interaction, *F*(2,26) = 4.86, *p* = 0.04; central, *p* = 0.79; centroparietal, *p* < 0.03; parietal, *p* < 0.05]. The distribution of the effect is shown in Figure 4. No differences between Welsh and English targets were detected during this time window in midline analyses (all *p*’s > 0.22).

**Figure 5:**
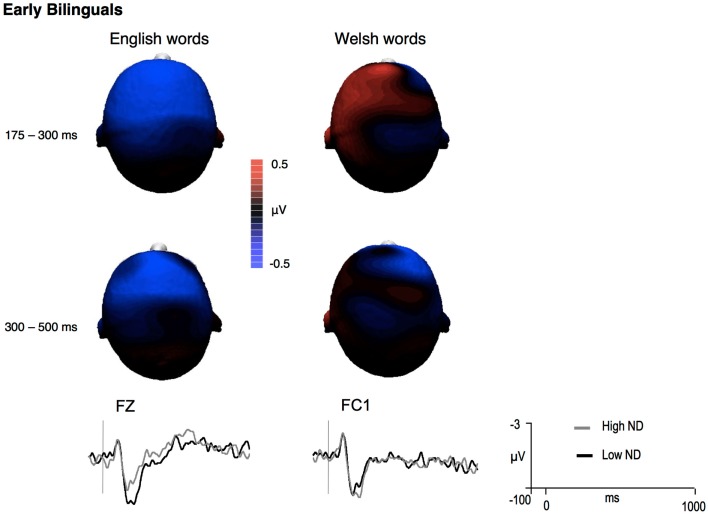
**Difference voltage maps representing the cross-language ND effect (high ND – low ND) in early bilinguals and mean grand-averages ERPs at the site where the effect was largest (only for the 175–300 ms time window, as no significant cross-language ND effects were observed during the 300–500 ms time window; negative is plotted up)**.

Hemisphere analyses over frontal and frontocentral sites revealed a trend for the interaction between Language and CLND [*F*(1,13) = 3.8, *p* = 0.07]. The ND effect tended to be significant for English targets [*F*(1,13) = 4.1, *p* = 0.06]. For Welsh targets, ND interacted with Hemisphere [*F*(1,13) = 4.68, *p* = 0.05]. Over the left hemisphere, the ND effect was reversed, in that Welsh words with high CLND tended to elicit more positive ERP amplitudes than Welsh words with low CLND [*F*(1,13) = 4.25, *p* = 0.06]. No ND effects were observed over the right hemisphere sites (all *p*’s > 0.29). The distribution of the effects is shown in Figure 5.

Midline analyses revealed a significant interaction between Language and CLND [*F*(1,13) = 4.86, *p* < 0.05]. The ND effect was significant for English [*F*(1,13) = 5.00, *p* < 0.05] but not for Welsh (*p* = 0.39) targets.

#### 300–500 ms

In hemisphere analyses, a trend was found for the interaction between ND, Hemisphere, and Laterality [*F*(1,13) = 4.2, *p* = 0.06], suggesting distributional differences between high and low CLND targets. However, no ND effects resulted significant in follow-up analyses. No ND or Language effects were detected in this time window in midline analyses (all *p*’s > 0.21).

## Discussion

The present study was aimed at replicating and extending Midgley et al.’s (2008) data on the effects of CLND in visual word recognition by comparing late and early bilinguals. Late bilinguals learned Welsh later in life, whereas early bilinguals were exposed to both English and Welsh either at birth or during early childhood. Both behavioral and electrophysiological data revealed differences between the two languages in late bilinguals: they were less accurate and slower in detecting Welsh targets compared to English targets in the categorization task; furthermore, Welsh words elicited more negative ERPs than English words starting at around 300 ms, suggesting that L2 words required more processing resources than L1 words. These large effects were absent in early bilinguals, who only showed slower RT to Welsh than English targets in the categorization task, likely reflecting the fact that English remained, in terms of reading, the dominant language. Electrophysiologically, only a small (0.2 μV) effect was found over centroparietal and parietal sites, where English targets elicited more negative ERP amplitudes compared to Welsh targets in the 175–300 ms time window.

As expected, based on Midgley et al. (2008), targets with high CLND elicited more negative ERPs as compared to low CLND targets over central, centroparietal, and parietal sites from 175 to 500 ms in late bilinguals. In contrast to Midgley and colleagues, this effect did not interact with Language, implying that both English and Welsh targets contributed to it. Therefore, in proficient late bilinguals, words in one language activate the orthographic representation of words in the other language before 250 ms, supporting the non-selective access account of single word recognition (e.g., Dijkstra and Van Heuven, 2002, but see Wu and Thierry, 2010, for the case of low proficient bilinguals with languages very different in terms of script). According to this model, the two languages are integrated in a single lexicon; presentation of a word in a language causes the activation of words in the other language that overlap in form (orthographic and phonological) and/or meaning. Therefore, it is the similarity between the stimulus and internal representations that drives activation, not the language to which words belong (Dijkstra and Van Heuven, 2002). Indeed, the Language effect started later than the CLND effect in late bilinguals. Furthermore, given that L2 was opaque in Midgley et al. (2008) and transparent in the present experiment, we can conclude that CL orthographic neighborhood effects are not modulated by orthographic transparency, in line with data from studies on within-language ND.

The lack of interaction between Language and ND in the 175–300 ms time window might have been due to the small number of participants. Inspection of Figure 3 suggests that the effect was not completely symmetrical (analyses run on each language separately confirmed that the ND effect was significant for Welsh but not English targets: Welsh, *p* < 0.04, English, *p* = 0.67 in hemisphere analyses; Welsh, *p* = 0.005, English, *p* = 0.16 in midline analyses). We asked whether differences in experience and proficiency with L2 among our participants might have contributed to this pattern of results. Our participants were, as a group, highly proficient, considering their performance in the translation and categorization tasks (a few scored nearly at, or at, ceiling). However, differences in proficiency and experience existed among them (for example, accuracy in the translation task ranged from 48 to 100%). Furthermore, they reported using Welsh for recreational reading 22.5% of the time (only two participants reported reading Welsh 50% of the time). It is therefore possible that even many years of experience with a second language do not translate in completely symmetrical effects in reading experiments if the first language remains dominant, particularly here in the domain of reading (which is certainly the case for most English-Welsh bilinguals, given that Welsh is a “minority” language in Wales; Lyon, 1996). The Language effect, along with the behavioral results, supports this picture.

In order to assess whether the difference in ERP amplitude between high and low CLND English (L1) targets was modulated by the participants’ experience with L2, *post hoc* analyses were carried out based on a median split with Years of Experience with Welsh and Translation Accuracy as measures of experience. The results (see Table 2) revealed the presence of larger CLND effects for L1 words in more, compared to less, proficient bilinguals for both the early and late time window, as expected: more experienced bilinguals were supposed to have a broader Welsh vocabulary, likely including many Welsh words that were neighbors of English targets in the present study. These findings suggest that CLND had some effect on the processing of L1 words, depending on the experience with the second language. This pattern is in agreement with non-selective access models, given that CL neighbors are hypothesized to be activated differentially based on a variety of factors that affect the level of activation of single items, such as subjective frequency and proficiency in the second language.

**Table 2:** **Cross-language ND effects for English targets in late bilinguals in terms of effect size (differences are in μV)**.

Years of experience	Time window (ms)	Less experienced bilinguals	More experienced bilinguals	Cohen’s *d*
	175–300	−0.08 (1.37)	−0.59 (0.77)	0.60*
	300–500	0.03 (1.27)	−0.32 (0.70)	0.34^††^
Translation accuracy	Time window (ms)	Lower accuracy translators	Higher accuracy translators	Cohen’s *d*
	175–300	−0.14 (1.43)	−0.38 (0.80)	0.21^†^
	300–500	−0.03 (1.28)	−0.26 (0.70)	028^†^

*Medium effect; ^††^, small-to-medium effect; ^†^ small effect. The effects were calculated at CPZ, where the cross-language ND effect was largest (SD are shown in parentheses). The median split for Years of Experience with Welsh was 12 years (the less experienced group had an average of 6.7 years, whereas the more experienced group had an average of 17 years). The median split for Translation Accuracy was 83% (the lower accuracy translators had an average translation accuracy of 69%; the higher accuracy translators had an average translation accuracy of 92.6%).

As in Midgley et al. (2008), the interaction between CLND and Language was significant in the 300–500 ms time window in late bilinguals. The effect was significant for L2 targets but not for L1 targets, revealing asymmetric effects for the two languages. Therefore, the early activation of Welsh neighbors when participants read English words might have dissipated rapidly and did not carry out to the N400 time window. Median split *post hoc* analyses based on language proficiency suggest the presence of larger CLND effects for L1 words in more, compared to less, proficient bilinguals, as for the 175–300 ms time window. Overall, these results suggest that, in late bilinguals, electrophysiological CLND effects tend to be asymmetrical, although the level of asymmetry was modulated by experience with the second language, in agreement with behavioral data (e.g., Bijeljac-Babic et al., 1997).

Based on the non-selective access hypothesis, it was hypothesized that symmetrical effects would be present for the two languages in early bilinguals. However, this hypothesis was not supported. A frontocentral CLND effect was found at midline sites for English targets in the 175–300 ms time window. For Welsh targets, the effect was mainly localized over the frontal left hemisphere sites and it was reversed. Perhaps high CLND Welsh words (e.g., *bara*, *coes*, *nain*) automatically activated competing English phonological representations, which would cause inhibition (e.g., Dijkstra et al., 1999); but it is unclear why this would occur only with Welsh targets and only in early bilinguals. Additionally, no CLND effects were found in the 300–500 ms time windows in early bilinguals. Therefore, CLND effects were weaker and more transient in early bilinguals. Furthermore, their pattern only partially resembled the one observed in late bilinguals in terms of distribution and direction. Although the meaning of these differences is unclear, the presence of effects in the 175–300 ms time window in early bilinguals reveals the existence of CL activation during the early stages of reading. It might be safe to conclude, based on the present data, that this activation is quickly suppressed or dissipated, potentially because the inhibitory control operating in early bilinguals is more efficient and has a faster turn around than that developed by late bilinguals.

These results are not entirely consistent with a non-selective model of lexical access, as they seem to contradict behavioral accounts of CL activation in bilinguals. However, most of the available data on CLND effects was gathered in proficient late bilinguals (e.g., Midgley et al., 2008) or participants whose age of acquisition for L2 was not specified (e.g., Grainger and Dijkstra, 1992; van Heuven et al., 1998), with a few exceptions. For example, Bijeljac-Babic et al. (1997) found CL activation of orthographically related words in early French-English bilinguals who learned both languages during early childhood and who used them daily. However, the masked priming paradigm employed by the authors is fairly different from the categorization task used in the present experiment, since, in the latter, the “context” language was known to the participants (while it was masked in Bijeljac-Babic and colleagues’ study). Therefore, early bilinguals might be skillful at applying top-down inhibition to block interference from words from the other language if the linguistic context is clear (e.g., Rodriguez-Fornells et al., 2002).

Electrophysiological evidence is mixed. In a letter detection task, Rodriguez-Fornells et al. (2002) asked early Spanish-Catalan bilinguals to respond to Spanish words presented singularly on a computer screen along with Spanish pseudowords and Catalan words and non-words (different response hands were used depending on the word’s initial letter). The authors found a N400 modulation by lexical frequency only for Spanish words and therefore hypothesized that proficient bilinguals are able to block semantic processing in the unattended language (for a critique of this work, see Grosjean et al., 2003). This conclusion contradicts more recent evidence of CL automatic semantic priming in early bilinguals. Martin et al. (2009) asked participants to indicate whether words presented on a computer monitor at regular intervals in a visual stream had more than five letters or five or fewer letters. This task was aimed at forcing participants to focus on the stimuli’s low-level features, instead of their meaning. Participants saw two blocks of trials, depending on whether they had to respond only to Welsh or English stimuli. They were not informed that words were presented in pairs, belonging to the same or different languages and being semantically related or unrelated. The results revealed that the N400 was modulated by the semantic relationship between primes and targets, regardless of whether the words belonged to the same language and regardless of whether they were in the language under the focus of attention. Martin and colleagues concluded that word meaning is accessed automatically for both languages in early bilinguals because it occurred even when participants were explicitly instructed to neglect words in a given language. According to them, the task was successful in driving the participants’ attention away from semantic processing, as no behavioral semantic priming effect was found in either experiment for reaction times. However, it is unclear why the activation of meaning would have any priming effect on a letter-counting task. Furthermore, the authors did not perform a manipulation check to establish that participants were indeed unaware of the semantic relationship between some of the words. Finally, as Martin and colleagues acknowledged (p. 330), in order to decide whether a stimulus required a manual response, attention needs to be paid to either its word form or meaning. Therefore, the very goal of having participants disregard words in one language might have caused them to engage in lexical and semantic processing of every word. This being said, Martin et al. (2012) recently showed that the same task in monolingual speakers of English failed to elicit any semantic modulation of the N400, even when participant focused on English words. Obviously, the critique of Martin et al. (2009) applies equally to Rodriguez-Fornells et al.’s (2002). Further research is needed to settle the question. In the meantime, the present results suggest that late and early bilinguals might exercise different levels of control on one language when processing words in their other language, at least as regards CL activation of orthographic neighbors.

The functional meaning of the differences in CLND effects between early and late bilinguals is not clear. Differences in proficiency alone are unlikely to explain this pattern, as targets in both languages contributed to the ND main effect in late bilinguals. Therefore, based on proficiency, a more symmetric pattern would be expected in early bilinguals^2^. Early and late bilingualism differ on a variety of dimensions. Early or childhood bilingualism (which itself can be distinguished in various forms, e.g., simultaneous and sequential) tends to occur in more naturalistic settings, while late bilingualism is usually fostered through direct instruction and often without a relevant pragmatic context (Baker, 2011). Furthermore, because early bilinguals usually learn to speak their languages in different contexts and with different people, they develop an awareness of the distinct use of different languages and two separate language systems very early (Baker, 2011). This original and reciprocal independence might help set up control mechanisms that are not present in late bilinguals. Whilst speculative, this hypothesis highlights the fact that current models of non-selective access (e.g., the BIA+) do not take into consideration differences in learning experiences that often characterize language acquisition in early and late bilinguals.

The results observed in late bilinguals support the recent literature on the modulation of the N400 amplitude by ND (Holcomb et al., 2002; Midgley et al., 2008; Müller et al., 2010; Laszlo and Federmeier, 2011). They also support the presence of early ND effects in CL experiments starting at around 175–200 ms. Interestingly, early effects have not been reliably described in studies of within-language ND, with the exception of Holcomb et al.’s (2002) second experiment. Midgley et al. (2008) explained this apparent discrepancy in terms of differences in the relative frequency of targets and their neighbors in within- and between language ND studies: in the latter, L2 targets might have L1 neighbors with higher subjective frequencies, compared to L1 neighbors of L1 targets. This relative frequency would translate in an earlier influence of the neighbors on the processing of the target word. This reasonable explanation, however, does not account for the presence of early effects in Holcomb et al.’s (2002) second experiment. Furthermore, the ND effects in Laszlo and Federmeier (2011) seemed to start earlier than 250 ms, based on their Figure 3, although the authors limited their analysis to the 250–450 ms time window. Similarly, Müller et al.’s (2010) Figure 2 suggests the presence of an early effect; however, the authors concentrated their analyses on the 350–550 and later time windows. Clearly, the presence of early within-language ND effects will need to be substantiated in future experiments. In the meantime, we would recommend that analyses be carried out on earlier time windows, as both Midgley et al.’s study and the present findings suggest that ND effects are detectable before 300 ms.

One of the limitations of the present study is the relatively small sample size. Future studies should investigate individual differences more systematically, as the present data suggest that the presence of CLND effects for L1 depend on proficiency in L2, at least in late bilinguals. Furthermore, future studies should investigate how different language learning experiences shape aspects of cognition and brain organization in terms of CL interaction, as the differences between early and late bilinguals are not trivially explained by non-selective models of lexical access.

## Conflict of Interest Statement

The authors declare that the research was conducted in the absence of any commercial or financial relationships that could be construed as a potential conflict of interest.

## Footnotes

^1^The data discussed in this paper are from the same study described in Grossi et al. (2010); in that paper, we focused on the N1 lateralization for the two languages; here, we focused on cross-language neighborhood effects, investigated in different time windows.

^2^However, some authors have remarked that cross-language effects tend to be larger when the target words in one language have a lower frequency than related words in the other language (e.g., Dijkstra et al., 1999; p. 497; see also Beauvillain and Grainger, 1987). Therefore, based on written frequency or familiarity, larger cross-language effects would be expected for L2 targets in late bilinguals, and smaller effects would be obtained in early bilinguals, for whom words in the two languages have more similar frequencies.
